# Hilar Somatostatin Interneurons Contribute to Synchronized GABA Activity in an *In Vitro* Epilepsy Model

**DOI:** 10.1371/journal.pone.0086250

**Published:** 2014-01-21

**Authors:** Sabine Grosser, Bridget N. Queenan, Rupa R. Lalchandani, Stefano Vicini

**Affiliations:** 1 Institute of Neurophysiology, Charité Universitätsmedizin Berlin, Germany; 2 Department of Psychiatry and Psychotherapy, Charité Universitätsmedizin Berlin, Germany; 3 Department of Pharmacology and Physiology, Georgetown University, Washington, District of Columbia, United States of America; 4 Interdisciplinary Program in Neuroscience, Georgetown University, Washington, District of Columbia, United States of America; 5 Graduate Program in Physiology and Biophysics, Georgetown University, Washington, District of Columbia, United States of America; McGill University, Canada

## Abstract

Epilepsy is a disorder characterized by excessive synchronized neural activity. The hippocampus and surrounding temporal lobe structures appear particularly sensitive to epileptiform activity. Somatostatin (SST)-positive interneurons within the hilar region have been suggested to gate hippocampal activity, and therefore may play a crucial role in the dysregulation of hippocampal activity. In this study, we examined SST interneuron activity in the *in vitro* 4-aminopyridine (4-AP) model of epilepsy. We employed a multi-disciplinary approach, combining extracellular multi-electrode array (MEA) recordings with patch-clamp recordings and optical imaging using a genetically encoded calcium sensor. We observed that hilar SST interneurons are strongly synchronized during 4-AP-induced local field potentials (LFPs), as assayed by Ca^2+^ imaging as well as juxtacellular or intracellular recording. SST interneurons were particularly responsive to GABA-mediated LFPs that occurred in the absence of ionotropic glutamatergic transmission. Our results present evidence that the extensive synchronized activity of SST-expressing interneurons contribute to the generation of GABAergic LFPs in an *in vitro* model of temporal lobe seizures.

## Introduction

Temporal lobe epilepsy is the most common type of adult pharmacoresistant focal seizure disorder, characterized by excessive and abnormally synchronous activity in the hippocampus and surrounding cortex [1]. GABAergic interneurons of the hippocampal hilus are thought to act as a gate for runaway excitation [2], and have therefore been implicated in the pathogenesis of temporal lobe epilepsy. The two major subtypes of interneurons in this area are the parvalbumin (PV)-positive fast-spiking interneurons and the somatostatin (SST)-positive, low-threshold spiking interneurons [3]; [4]. As SST-interneurons are strongly implicated in gating hippocampal activity [2], we investigated the role of SST-expressing interneurons in the generation of epileptiform synchronization using mice that express Cre recombinase in this specific neuronal population [5].

The 4-aminopyridine (4-AP) model of epilepsy has been used extensively to investigate epileptiform activity *in vitro* [6]–[16]. Using perforated multielectrode array (pMEA) recordings, we have previously found that 4-AP induces distinct classes of local field potentials (LFP), which differ in the location and nature of origin: while “brief” interictal LFPs originate predominantly in the CA3 pyramidal layer, “longer lasting” LFPs can be generated in the different areas of the hippocampus [9], [15]. Blockade of excitatory synaptic transmission reveals that the “longer lasting” LFPs are generated by the synchronous activity of GABAergic interneurons in the dentate gyrus and hilar region of the hippocampus [7]–[10]; [15]–[16].

Using a multidisciplinary approach that combined extracellular and intracellular recording with optical imaging, we studied the activity of SST interneurons during epileptiform activity. We used Cre recombinase-driven expression of the GCaMP3 optical Ca^2+^ sensor [17] in SST-Ires-Cre neurons [5] to selectively express GCaMP3 in SST-positive interneurons. Combining this optical imaging with extracellular recordings using pMEA and patch-clamp recordings from visually identified SST- interneurons, we found that SST interneurons are strongly synchronized during all LFPs. We also found that SST interneurons are driven more extensively by neuronal activity resulting from the combined activation of dentate granule (DG) granule cells and CA3 pyramidal neurons. Although SST- interneurons all behaved similarly during 4-AP-induced epileptiform activity, upon blockade of glutamatergic transmission we revealed distinct action potential firing patterns of these neurons, which might be related to the generation of long-lasting, GABA-mediated LFPs [9].

## Materials and Methods

### Transgenic Animals

SST-Ires-Cre mice (Ssttm2.1(cre)Zjh/J, Jackson Lab strain # 013044, [5] were crossed with Rosa-tdTomato reporter (B6;129S6-Gt(ROSA)26Sortm9(CAG-tdTomato)Hze/J Jackson lab Strain # 007905, [18]or GCAMP33 reporter (B6;129S-Gt(Rosa)26Sor<tm38<CAG-GCAMP33)Hze>/J Jackson lab Strain # 014538, mice, [17]. The resulting offspring displayed the Rosa-tdTomato or the GCAMP3 proteins, respectively, in the Cre-expressing neurons. Genotyping was performed with a commercial vendor (Transnetyx, Cordova, TN).

### Slice Preparation

All experiments were performed in accordance with the Georgetown University Animal Care and Use Committee (GUACUC) and in accordance with the National Institutes of Health Guide for the Care and Use of Laboratory Animals (NIH Publications No. 8023, revised 1978). The protocol was approved by the GUACUC (Permit Number: 10-055). Efforts were made to minimize animal suffering and the number of animals used. Mice at postnatal day (p) 13–24 were decapitated and brains were removed quickly into ice cold cutting solution containing (in mM): 86 NaCl, 3 KCl, 4 MgCl_2_, 1 NaH_2_PO_4_, 75 sucrose, 25 glucose, 1 CaCl_2_, and 25 NaHCO_3_, at pH 7.4. Horizontal slices of 275 µm thickness were prepared (Vibratome 3000 Plus Sectioning System, Vibratome, St. Louis, MO) preserving the hippocampal structure. Slices were recovered in pre-warmed artificial cerebrospinal fluid (aCSF, 34°C) containing (in mM): 124 NaCl, 2.5 KCl, 1 MgCl_2_, 10 glucose, 1 CaCl_2_, and 26 NaHCO_3_ for 30 minutes, and stored in room temperature aCSF until use. All solutions were constantly aerated with a 95% O_2_ and 5% CO_2_ gas mixture. Chemicals were acquired from Sigma-Aldrich (St Louis, MO).

### Pharmacology

Spontaneous epileptiform activity was induced by adding 4-Aminopyridine (4-AP, 100 µM) to the recording solution. Ionotropic receptor antagonists were used to examine single unit activity in the dentate gyrus and CA3 regions in the absence of synaptic transmission. Glutamatergic transmission was suppressed by concurrently applying both the N-methyl-D-aspartic acid (NMDA) receptor antagonist 3,3-(2-carboxypiperazine-4yl)propyl-1-phosphonate (CPP, 20 µM) and the 2-amino-3-(5-methyl-3-oxo-1,2-oxazol-4-yl)propanoic acid (AMPA) receptor antagonist 2,3-dihydroxy-6-nitro-7-sulfamoyl-benzo[f]quinoxaline-2,3-dione (NBQX, 20 µM). Solutions were perfused over the slice by gravity. Chemicals were acquired from Abcam Inc. (Cambridge, MA).

### pMEA Recordings

Neuronal network activity was recorded using a perforated multi-electrode array (pMEA, Multi Channel Systems, Reutlingen, Germany) with 59 embedded titanium nitrite electrodes, each with a diameter of 30 µm and an inter-electrode spacing of 200 µm. Recorded electrical activity from each channel was digitized at a sampling rate of 5 kHz and acquired using a MEA2100 amplifier and MC Rack 4.0 software (Multi Channel Systems, Reutlingen, Germany). The temperature in the recording chamber was constantly monitored using an analog YS1 Thermometer (Yellow Spring Instruments, Yellow Springs, OH) and maintained at 26 to 28°C by a heated perfusion cannula (ALA Instruments, Farmingdale, NY).

### Single and Dual Patch-clamp Recordings

Single- and dual-cell patch-clamp recordings were performed from visually identified fluorescent SST-positive interneurons in the hilar region of the dentate gyrus in 16 slices derived from 10 mice using a fluorescent microscope (Axioskop, Zeiss, Jena, Germany). Juxtacellular, cell attached, recordings to record action potentials firing rates from outside the membrane without breaking in the cell were performed from 22 SST-interneurons and whole cell recordings after breaking the membrane to evaluate action potentials and of synaptic activity from 16 SST- interneurons.Patch-clamp microelectrodes were fabricated from borosilicate capillary tubes and pulled using a two-step pipette puller (PP-83, Narishige, and Tokyo, Japan). Pipettes with tip resistances between 4–6 MΩ were filled with internal solution (in mM): 145 K-gluconate, 1.1 EGTA, 5 MgATP, 0.2 NaGTP, and 10 HEPES, adjusted to pH 7.2 with KOH. Recordings were performed using Axopatch 1D and Axopatch 200B amplifiers, a Digidata 1440A interface, and pClamp software (all from Molecular Devices, Sunnyvale, CA). Signals were low-pass filtered at 2kHz, sampled and processed at 5 kHz, and acquired with two analog channels of the MCS amplifier MEA-2100. Data are shown as mean ± SEM.

### Imaging

**Figure 1:**
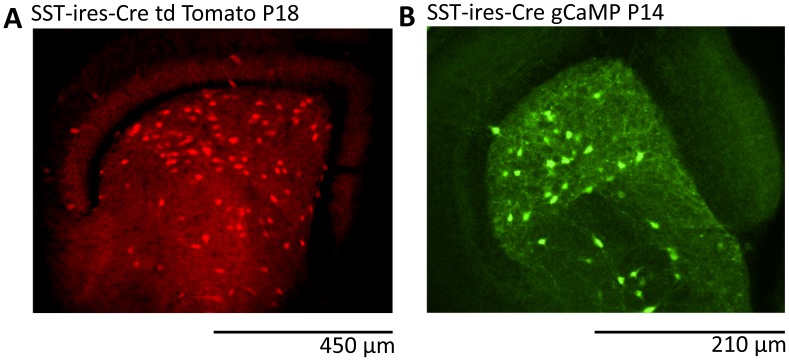
Distribution of somatostatin-positive interneurons in the hilus of SST-Ires-Cre mice. Representative fluorescence micrographs (Z-projection of confocal stacks) illustrating the expression of td-Tomato (**A**) and GCaMP3 (**B**) in the hippocampal hilus of SST-Ires-Cre reporter mice (postnatal days 18 and 14, respectively). GCaMP3 imaging was performed in the presence of 40 mM KCl to achieve maximal activation of the Ca^2+^ sensor.

To determine the maximal distribution of GCaMP3 expressing neurons, confocal fluorescence imaging of hippocampal slices from SST-GCaMP3 mice was performed in the presence of 40 mM KCl (**Figure 1B**). For all other experiments, cells were imaged in aCSF using a fluorescent microscope (Axioskop, Zeiss, Jena, Germany) equipped with Plan-Neofluar 2.5x/0.075, N-Achroplan 20x/0.5, and N-Achroplan 40x/0.75 objectives (Zeiss, Germany). Images were captured at a sampling rate of 20 Hz using a charge-coupled device camera (CoolSNAP HQ^2^, Photometrics, Tucson, AZ) and Nikon Elements software (Nikon, Japan). Calcium responses were recorded using the intrinsic fluorescent properties of the fluorescent Ca^2+^ sensor in the SST-interneurons following 488 nm centered wavelength excitation (PhotoFluor II, 89 North, Burlington, VT). To monitor the changes of the calcium signal, image sequences were taken every 50 ms for a period of 2 minutes and analyzed using ImageJ (National Institutes of Health, Bethesda, MD). After background subtraction, regions of interest (ROI) were selected manually and changes in intensity levels (ΔF/F) over time were computed for each ROI with Clampfit (Molecular Devices, Sunnyvale, CA).

## Results

In this study we took advantage of a combination of imaging, patch-clamp recordings and multi-electrode array recordings to determine the activity of the SST neurons, an important subclass of GABAergic interneurons in the hippocampus, during the 4-AP model of epileptiform activity. We used SST-Ires-Cre mice [5] to drive the Cre recombinase-driven expression of either the red fluorescent reporter td-Tomato (**Figure 1A**) or the calcium sensor GCaMP3 (**Figure 1B**) in SST- interneurons. Within the hippocampus, the hilus contained a high number of SST- interneurons (**Figure 1**), consistent with previous reports [5], [19]. The presence of a fluorescent probe allowed us to perform patch-clamp recordings from visually identified interneurons, while recording extracellular activity throughout the slice with a perforated multi-electrode array (pMEA).

**Figure 2:**
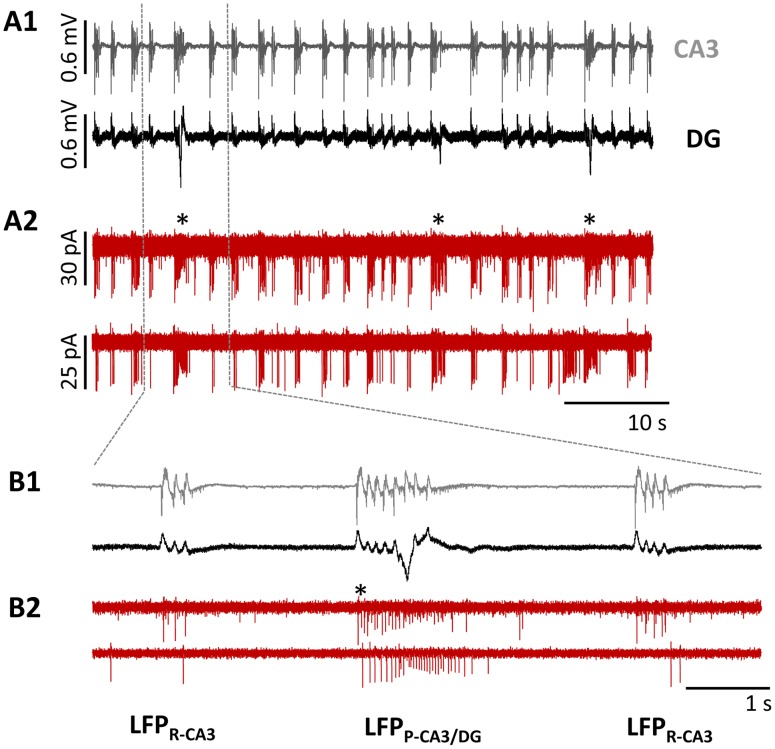
Firing of SST interneurons during 4-AP induced LFPs. Representative recordings of 4-AP-induced LFPs (A1,B1) and SST interneuron firing (A2,B2) during epileptiform activity. 4-AP provoked two classes of LFPs, detectable in CA3 (gray) and DG (black) hippocampal regions via extracellular pMEA recording (A1,B1): LFP_R-CA3_ and LFP_P-CA3/DG_ (indicated with *). Juxtacellular patch-clamp recordings (A2,B2) from two visually identified SST- interneurons demonstrate increased AP firing of SST-interneurons in correspondence to distinct LFPs. Sections of the traces in **A** are shown at an expanded time scale in panel **B**.

We used the previously characterized 4-AP model to induce epileptiform activity in hippocampal slices [15], [16] (**Figure 2**). Consistent with our previous reports [16], we observed two distinct classes of LFPs, the first originating and remaining for the large part in the CA3 region (CA3-restricted LFPs; LFP_R-CA3_) and the second propagating from CA3 to the DG (LFP_P-CA3/DG,_ indicated with asterisk in **Figure 2A** **1, B1**). We performed juxtacellular recordings from 22 SST-interneurons to compare the firing of individual SST neurons to these two classes of LFPs. SST- interneurons increased action potential firing in response to both types of LFPs, with the majority of neurons (12/16) showing more pronounced responses to the LFP_P-CA3/DG_. (**Figure 2A** **2, B2)**. Three neurons had comparable firing frequencies with both LFP_P-CA3/DG_ and LFP_ R-CA3_ and one neuron showed an increase in its action potential frequency only with LFP_ R-CA3_.

**Figure 3:**
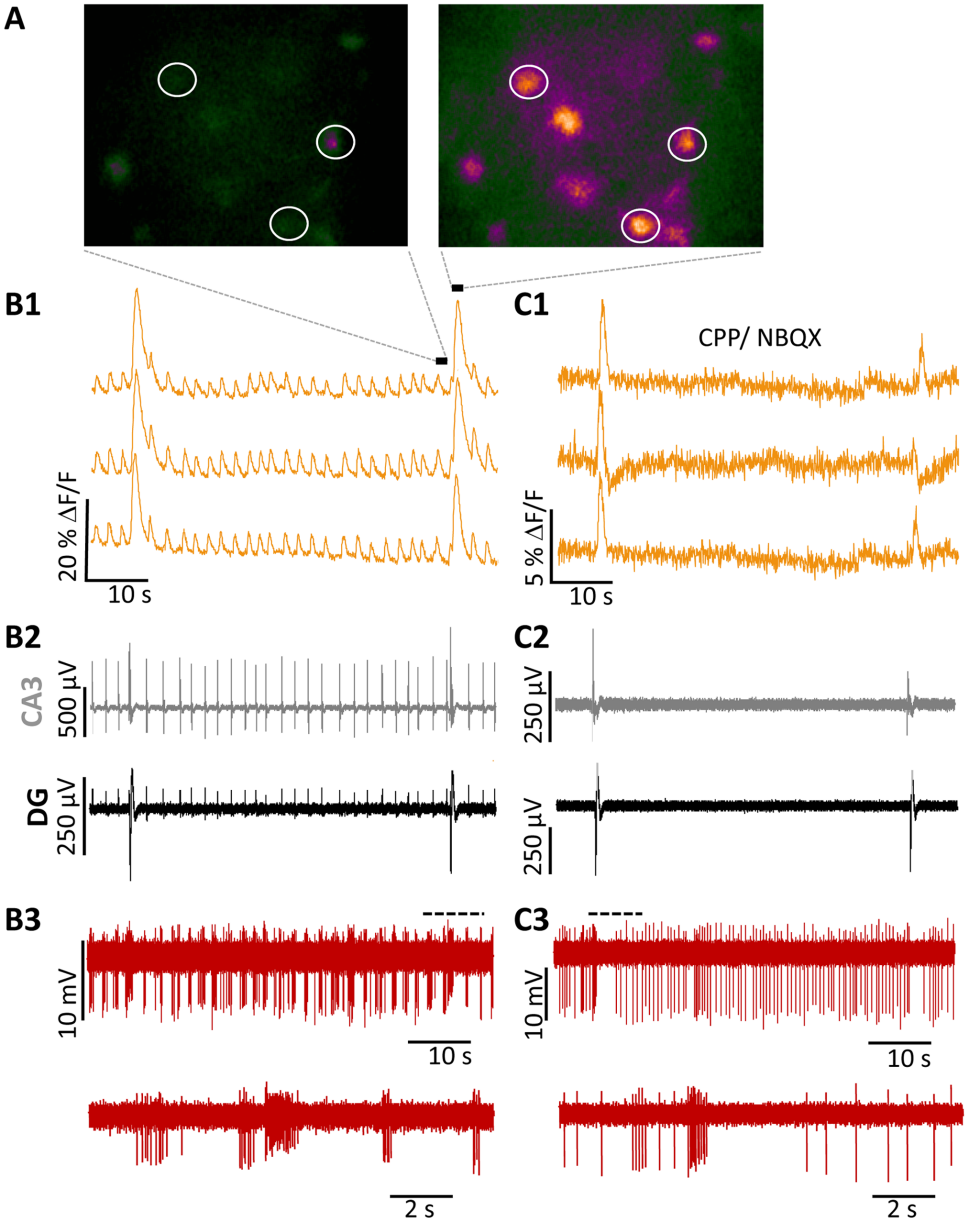
4-AP-induced LFPs provoke AP firing from SST-interneurons. (**A**) Representative images of GCaMP3 signal in a visual field containing several SST interneurons before (left) and during (right) LFP_P-CA3/DG_. (B) Simultaneous optical (B1) and juxtacellular (B3) recordings of SST activity during hippocampal LFPs (B2). B1: Fluorescent Ca^2+^ signals (ΔF/F) recorded from the ROIs indicated in (**A**). B2: Distinct LFPs recorded from pMEA electrodes located in the DG (black) and the CA3 (grey) regions.B3: Simultaneously recorded juxtacellular trace from a visually identified SST interneuron. (**C**) Same recording configuration as in (**B**) in the presence of the iGlurR antagonists, CPP and NBQX. Sections of traces in B3 and C3 (dotted line) are expanded below.

We also observed increased fluorescence signal with respect to LFPs using optical imaging using the genetically encoded Ca^2+^ sensor, GCaMP3 (**Figure 3A**). To confirm that the increased Ca^2+^ fluorescent corresponded to action potential firing, we performed juxtacellular recordings from SST- interneurons (**Figure 3B** **3**). Both GCaMP3 fluorescence intensity (ΔF/F) (**Figure 3B** **1**) and AP firing rate (**Figure 3B** **3**) increased in hilar SST interneurons during the LFP_P-CA3/DG_ and the LFP_ R-CA3_. However, the ΔF/F was consistently larger in correspondence to the LFP_P-CA3/DG_ than to the LFP_R-CA3_ (LFP_R-CA3_∶8.1±0.01%, N = 122 neurons; LFP_P-CA3/DG_: 23.2±0.05%, N = 46 neurons; Average LFPs in neurons pooled from 9 slices, p<0.05, paired t test).

To probe the origins of the distinct LFP classes, we blocked ionotropic glutamatergic receptor (iGluR) transmission using the NMDA- and AMPA-receptor antagonists, CPP (20 µM) and NBQX (20 µM), respectively (**Figure 3C**). As previously reported [15], [16], iGluR blockade decreased the frequency and amplitude of the small, interictal LFPs, revealing a large, drug-resistant LFP (CPP/NBQX LFP) (**Figure 3C** **2**, middle). Perfusion of hippocampal slices with bicuculline methobromide (25 µM) to block iontropic GABAergic transmission abolished all activity (5 of 5 slices tested), as reported previously [15], [16].

We therefore investigated the role of the SST interneurons in the generation of the large, GABAergic CPP/NBQX LFP. Upon perfusion with iGluR blockers, GCaMP3 imaging revealed a Ca^2+^ transients matching the CPP/NBQX LFP (**Figure 3C** **1**) with the average ΔF/F of 4.8±0.01% (N = 39 neurons from 9 slices), a considerable drop from the average ΔF/F of the LFP_P-CA3/DG_ (see above). In 12 of 22 SST- interneurons, iGluR blockade induced spontaneous action potential firing (3.81±0.76 Hz) in the absence of mean activity recorded with LFP as shown in the example in Fig. 3C bottom. In 9 of these neurons, action potential firing briefly increased at the onset of the CPP/NBQX LFP (9.6±2.2 Hz), followed by a silent period of variable length (**Figure 3C** **3**). In three other interneurons action potential firing was present only in correspondence with the CPP/NBQX LFP. The remaining 10 cells did not increase basal firing frequency and did not respond or had occasional spikes correlated with the CPP/NBQX LFP.

**Figure 4:**
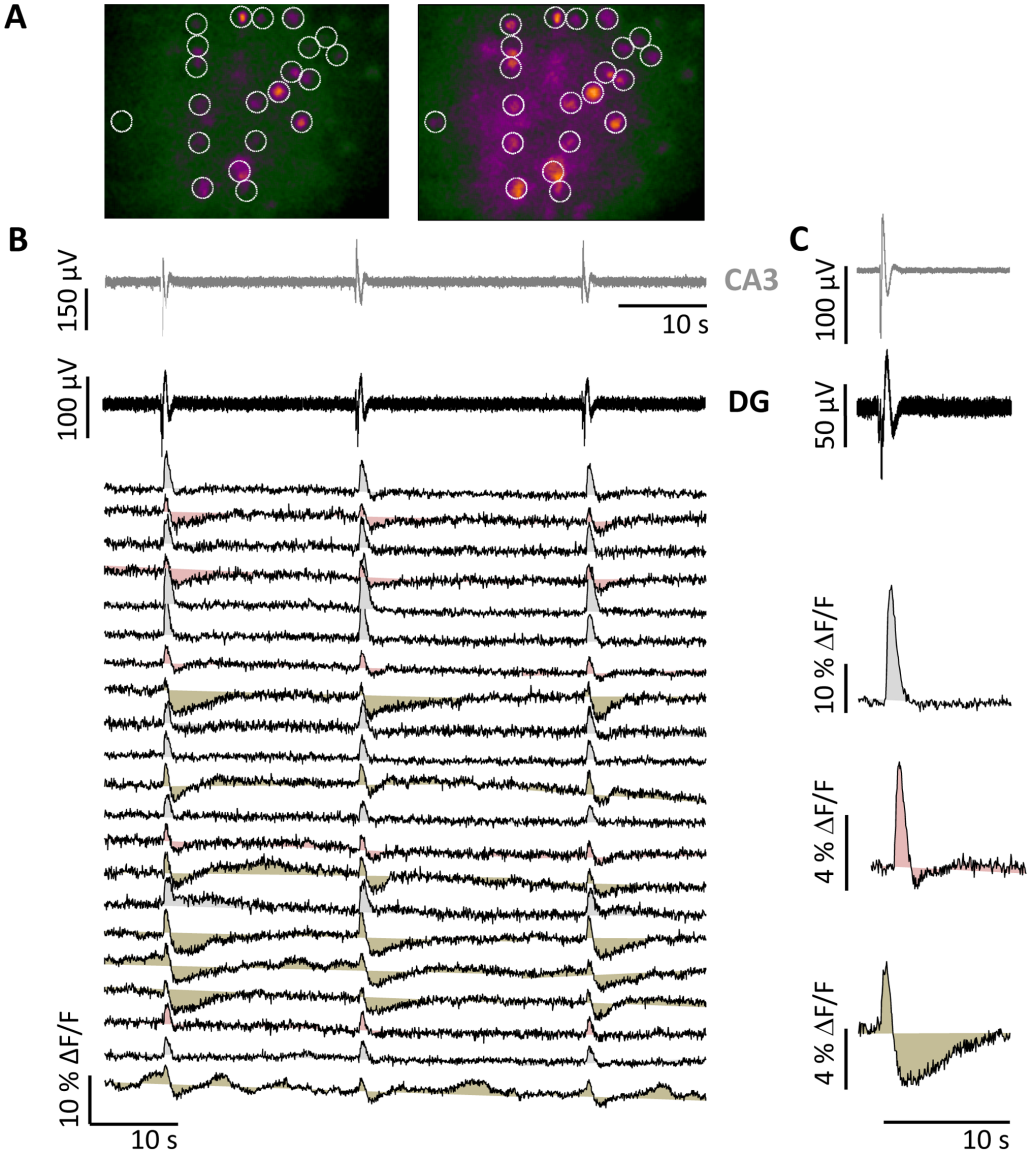
Variability of the GCaMP3 fluorescence response in SST interneurons during 4-AP induced LFPs. (A) Fluorescence image of GCaMP3-expressing SST interneurons before (left) and during a CPP/NBQX LFP (right). (B) Top: LFPs recorded in the presence of CPP/NBQX. Bottom: Ca^2+^ transients (ΔF/F) recorded from SST- interneurons in the presence of CPP/NBQX. Average change in ΔF/F triggered by the CPP/NBQX LFP reveals two distinct types of changes in Ca^2+^ transient in SST- interneurons. (C) Representative Ca^2+^ transients in three groups of SST- interneurons. Distinct monophasic (grey), intermediate (red) and biphasic (brown) Ca^2+^ transient are observed from SST- interneurons in response to CPP/NBQX LFPs recorded in DG and CA3 (top).

The Ca^2+^ transients evoked in SST- interneurons by the CPP/NBQX LFPs were often distinct between neurons in the same slice (**Fig. 4**). We observed three types of Ca^2+^ transients depicted in **Figure 4B–C**: i) cells with increased Ca^2+^ signal (grey fill), ii) cells with biphasic Ca^2+^ transients, consisting of an increased and then decreased Ca^2+^ signal (brown shade), or iii) cells displaying intermediate behavior (pink shade).

**Figure 5:**
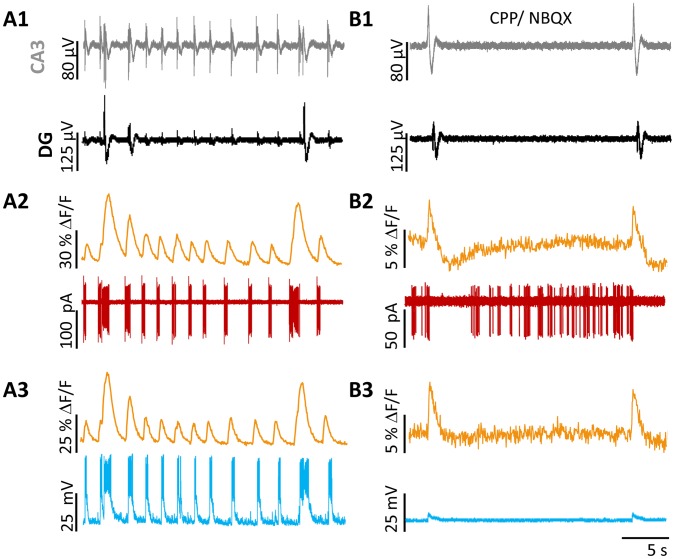
LFPs evoke spontaneous EPSPs with a large synchronous depolarization in SST- interneurons. GCaMP3 fluorescence (orange) recorded simultaneously with extracellular LFPs (A1) during juxtacellular (A2, red) and intracellular current-clamp (A3, blue) recording from two SST- interneurons. The depolarization and spike burst duration were longer in correspondence to the LFP_P-CA3/DG_ than to the LFP_R-CA3_ (A). Perfusion with CPP/NBQX (**B**) induced sustained spiking of the SST- interneuron with juxtacellular recording, but not for the SST-interneuron recorded in current clamp mode (resting membrane potential = −67 mV). Note the biphasic Ca^2+^ transient in cell #1 during CPP/NBQX application.

To gain insights into the events occurring in SST- interneurons during LFPs induced by 4-AP, we performed whole-cell recordings from visually identified neurons before and during glutamatergic blockade. GCaMP3 imaging again revealed consistently larger Ca^2+^ responses to the LFP_P-CA3/DG_ (**Figure 5A**, orange traces), corresponding to the increased AP firing observed in juxtacellular recordings (**Figure 5A** **2**). Intracellular current-clamp recordings revealed spontaneous PSPs with large synchronous depolarization during LFPs (**Figure 5A** **3**). We also performed simultaneous juxtacellular, whole cell, and MEA recordings together with Ca^2+^ signal. In 3 neurons from distinct mice, both the duration of the depolarization measured in current clamp recordings (1.2±0.1 s, n = 21 events), and the spike burst duration measured with juxtacellular recordings (1.3±0.8 s, n = 21 events) in correspondence to the LFP_P-CA3/DG_ were longer (p<0.05, unpaired t test) than those measured in correspondence to the LFP_R-CA3_ (0.28±0.12 s and 0.29±0.14 s, n = 65 events), suggesting a stronger activation of SST- interneurons during the LFP_P-CA3/DG_. In two of these neurons, perfusion with CPP/NBQX induced sustained spiking in the SST interneuron recorded in juxtacellular mode (**Figure 5B** **2**), although no such firing was observed in current-clamp mode (**Figure 5B** **3**). Interestingly, the long pauses in action potential firing seen in juxtacellular recordings after the CPP/NBQX LFP corresponded with a small, long-lasting synaptic potentials revealed in current clamp recordings,that was investigated further in voltage-clamp recordings (see below). In some of the recorded cells as illustrated before, the Ca^2+^ transient observed in the juxtacellular recorded cell was clearly biphasic (**Figure 5B** **2**), suggesting that the increase and subsequent pause in the firing rate during the CPP/NBQX LFP are the underlying mechanism. The transient however, was observed even in cells were no spiking was detected either in juxtacellular or in current clamp recordings (n = 13). Interestingly, the long pauses in action potential firing seen in juxtacellular recordings after the CPP/NBQX LFP corresponded with a small, long-lasting synaptic potentials revealed in current clamp recordings (**Figure 5B** **3**), that were not investigated further.

**Figure 6:**
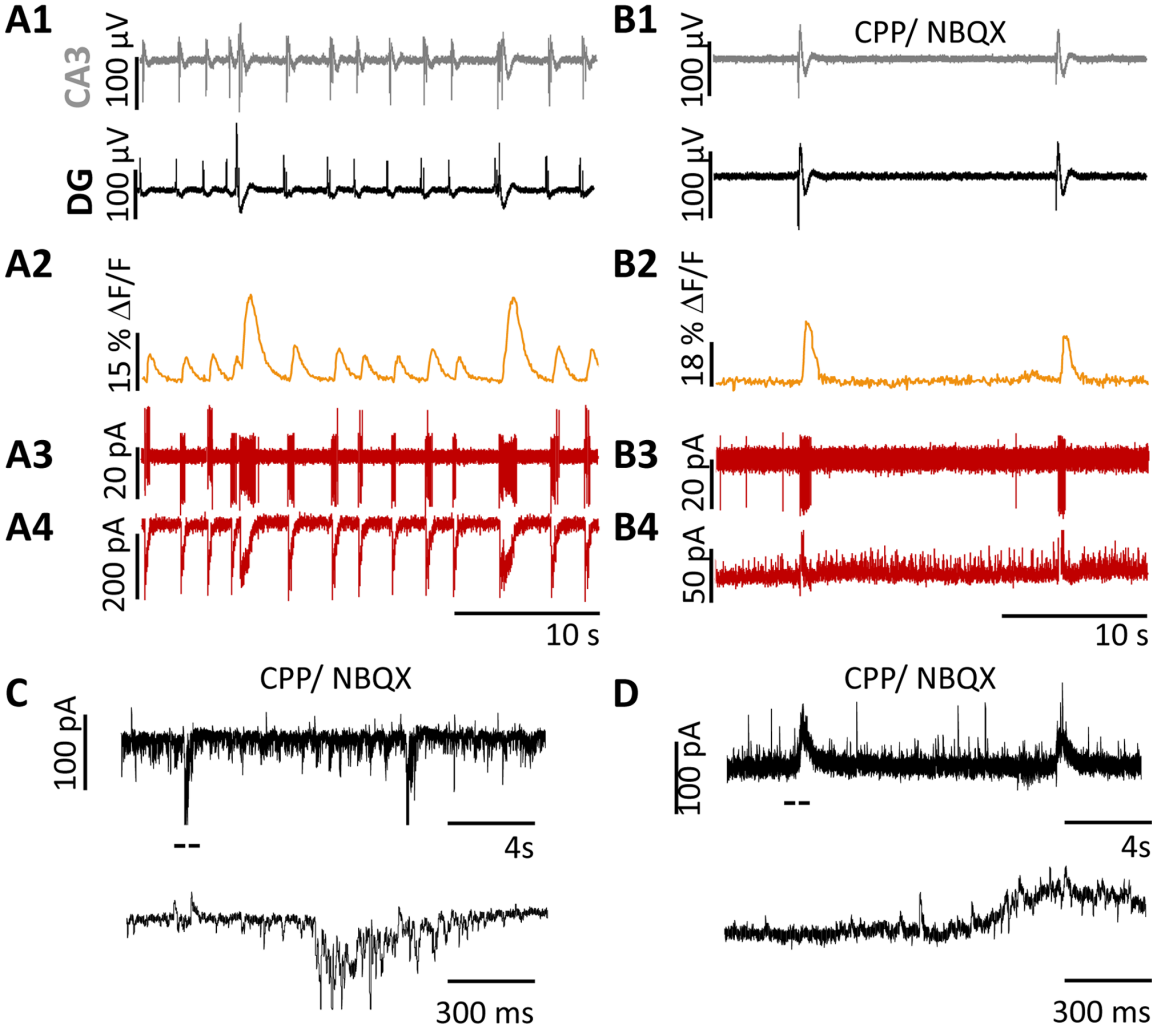
Voltage-clamp recordings of LFP-induced currents in SST- interneurons. (A) Extracellular pMEA recording of LFPs (A1) together with concurrent optical imaging (A2) and juxtacellular (A3) or voltage-clamp (A4) recording (Vh = −50 mV, holding current 45 pA) from two SST- interneurons. The inward currents observed were significantly longer and larger in correspondence to the LFP_P-CA3/DG_ than to the LFP_R-CA3_. Perfusion with NBQX/CPP (**B**) abolished the excitatory current revealing the occurrence of sustained IPSCs in association to the CPP/NBQX LFP. Note the increase in spontaneous IPSCs during the time between the LFPs occurrence. (**C**) Voltage-clamp recording (Vh = −52 mV, holding current 37 pA) from another SST- interneuron shown at different time scales illustrating the occurrence of inward EPSCs and less frequent outward IPSCs. Perfusion with NBQX/CPP (**D**) in the same neurons abolishes all EPSCs and reveals the occurrence of IPSCs.

To elucidate these findings, we compared optical (**Figure 6A** **2**) and electrical recordings in juxtacellular (**Figure 6A** **3**) and voltage-clamp mode at −50 mV (**Figure 6A** **4**). In this recording configuration we could observe at the same time EPSCs and IPSCs as inward and outward currents, respectively. The inward excitatory postsynaptic currents (EPSCs) 16 SST interneurons in 16 slices from 10 mice were on average significantly longer in correspondence to the LFP_P-CA3/DG_ but not larger, (duration: 1.23±0.14 s, amplitude: 391±90 pA, n = 21 events), than to the LFP_R-CA3_ (0.28±0.1 s, 190±42pA, n = 75 events; p<0.001 and p = 0.078 respectively, unpaired t-test versus LFP_P-CA3/DG_), suggesting that SST- interneurons receive a robust barrage of excitatory synaptic input from the hippocampal network during the LFP_P-CA3/DG_. In 9 neurons studied, perfusion with CPP/NBQX abolished the EPSCs corresponding to the LFP_P-CA3/DG_ and to the LFP_R-CA3_ (**Figure 6B**), revealing the occurrence of sustained outward inhibitory postsynaptic currents (IPSCs 47±21 pA, n = 23 events) in association with the CPP/NBQX LFP (**Figure 6C**). In addition, we observed a significant increase in spontaneous IPSCs frequency (from 1.09±0.04 Hz to 5.36±0.07 Hz) in the presence of iGluR blockers (**Figure 6B,D**), possibly deriving from spontaneous activation of other interneurons (see above and [16]).

## Discussion

Previous studies have suggested the occurrence of specialized hippocampal LFPs in the 4-AP model of epilepsy [7]–[10]; [15], [16]. In particular, there is strong evidence for “GABA-mediated LFPs” that occur synchronously in different areas of the hippocampus and in several other hippocampal subregions, [7]–[10]; [15], [16]. The application of iGluR blockers reveal that these events originate primarily in the DG area and that excitatory synaptic transmission is not required. Together, this suggests that synchronous activity of GABAergic interneurons is the likely underlying mechanism of these LFPs. Our findings bring experimental evidence to this hypothesis with a unique approach combining the strengths of pMEA, whole-cell patch clamp and Ca^2+^ imaging from genetically modified mice. We took advantage of the previously developed SST-Ires-Cre mouse model [5] to study SST interneurons tagged with fluorescent markers in the hilar region of the hippocampus. Using these methods, we present two novel findings. First, using juxtacellular recordings from SST- interneurons, we find that the majority of SST neurons fire more robustly with the LFP_P-CA3/DG_ than the LFP_R-CA3_, as recorded with pMEA electrodes in the DG and CA3. This result was extended to a larger number of neurons with the study of fluorescence changes in SST neurons. Secondly, our data suggest that the excitatory innervation of SST interneurons is strongly induced by neurons activated during the LFP_P-CA3/DG_, which include both CA3 pyramidal neurons and DG granule neurons. Together, these data bring evidence to the hypothesis that the synchronous activation of SST interneurons contributes to epileptiform activity.

SST interneurons were well synchronized with the two types of LFPs and little firing was seen in between LFPs. The combined application of two blockers of glutamatergic synaptic transmission increased the basal firing rate in over half of the SST neurons, resulting in pauses during the CPP/NBQX-induced LFP. This effect is likely due to the intrinsic firing properties of the neuropeptide Y (NPY)-expressing interneurons that hyperpolarize after bursts of activity [19] and have large overlap in the expression with SST interneurons [5], [19]. In some SST-interneurons however, we observed either no increase in basal firing or higher rates of basal firing with the CPP/NBQX LFP. This suggests that either the CPP/NBQX LFPs had a dual effect on these neurons or that two distinct populations of SST cells are present. The results of Ca^2+^ imaging further illustrate that there are various types of Ca^2+^ signaling in SST interneurons, with responses ranging from mono- to bi- phasic signaling. Additionally, combined data from pMEA, current clamp recordings and Ca^2+^ imaging indicate that small Ca^2+^ transients were present even without spiking of the recorded neuron. We speculate that dendritic calcium transients propagating to the soma might be the underlying cause of this effect. Nevertheless, this creates a limitation in the interpretation of the transients observed with Ca^2+^ imaging as they may not be exclusively attributable to spiking activity.

Our results demonstrate that in SST-interneuron, barrages of EPSCs are synchronized by the LFP_P-CA3/DG_ fields and the application of iGluR blockers uncovers IPSCs barrages. We also observed an increase in the baseline of spontaneous IPSCs (sIPSCs), which correlates with the increase in spontaneous firing observed from SST-interneurons in cell attach mode. Our study, however, failed to observe the progressive and larger shifts in holding current that one would expect if the proposed shifts in the extracellular K^+^ concentration were operative [10], [20]. The presence of strong IPSCs during the CPP/NBQX LFP suggest reciprocal innervation between SST-interneurons. This will explain the finding of distinct populations of SST interneurons, one that fire action potentials only during CPP/NBQX LFPs and the other that fire spontaneously and pause during the CPP/NBQX LFPs. This effect may be also related the rhythmic bursting of hilar NPY neuron evoked by 4-AP as shown in [19]. Our hypothesis of a reciprocal innervation between SST-interneurons would also imply that the balance of activity between the different populations of SST-interneurons might be involved in the mechanisms generating the CPP/NBQX LFP.

## Conclusion

Using multiple approaches, we present clear evidence to support the hypothesis that the synchronization of hilar SST GABAergic interneurons contribute to the generation of CPP/NBQX LFPs. While our findings do not expose the SST- interneurons to be the main players in the generation of these LFPs, we believe that other subtypes of interneurons may be the mastermind allowing for this synchronization. Further studies with other Cre-expressing mice may shed light on this important quest.
